# Supplementary figures and images for: REV3 promotes cellular tolerance to 5-fluorodeoxyuridine by activating translesion DNA synthesis and intra-S checkpoint
Source: PLoS Genet. 2024 Jul 2;20(7):e1011341. doi: 10.1371/journal.pgen.1011341 (PMC11249241; doi:10.1371/journal.pgen.1011341)

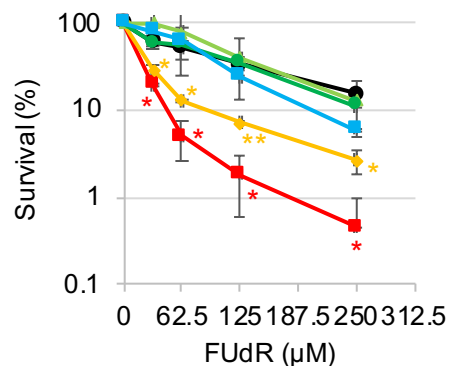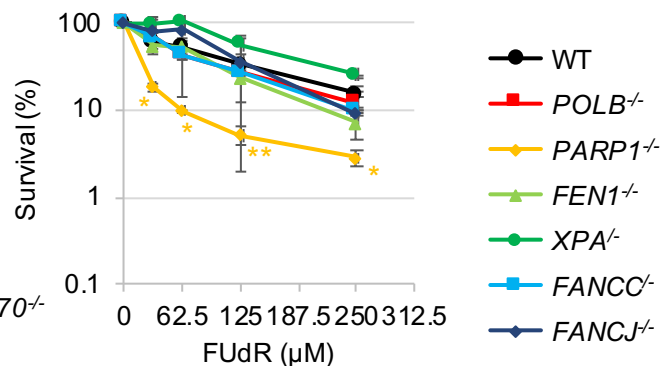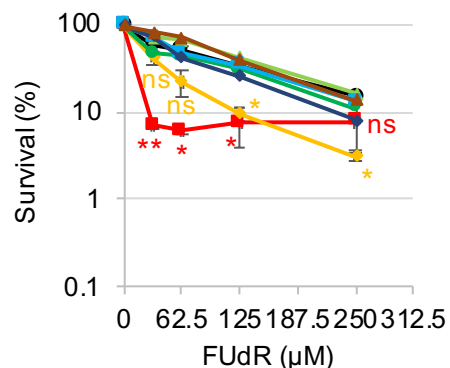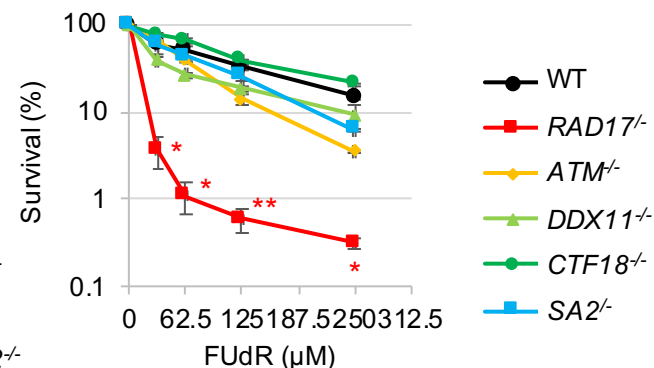

Supplement: S1 Fig — DT40 cells of the indicated genotypes were assessed for FUdR sensitivity. The cells were cultured for 48 h in the presence of FUdR at the indicated concentrations. The x-axis indicates the dose of FUdR on a linear scale, whereas the y-axis indicates the percentage of cell survival on a logarithmic scale. Error bars represent the standard deviation of three independent experiments. ns, not significant; *p < 0.05; **p < 0.01 (Student’s t-test). (PDF) [file pgen.1011341.s001.pdf]

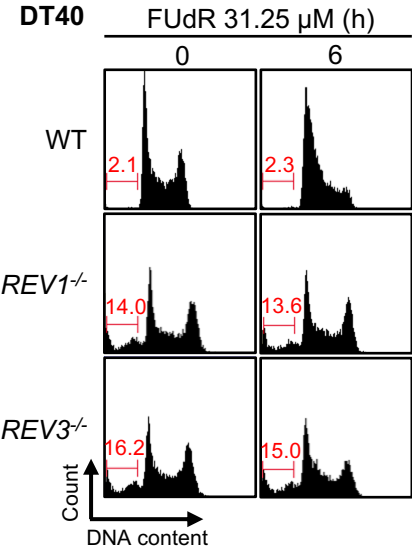

Supplement: S2 Fig — Indicated DT40 cells were treated with 31.25 μM FUdR for designated times (0–6 h). The histogram presents the cell cycle distribution. The DNA content (stained with propidium iodide) is displayed on the x-axis on a linear scale, and the number of cells counted is presented on the y-axis. Two peaks at 0 h correspond to cells in the G1 (left) and G2 (right) phases. The numbers above brackets represent the percentage of cells in sub-G1 (dead cell fraction). (PDF) [file pgen.1011341.s002.pdf]

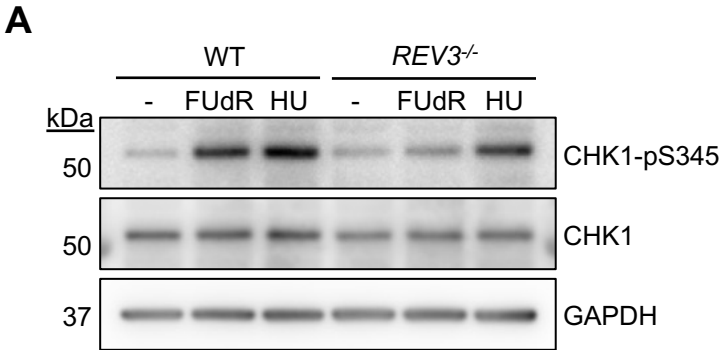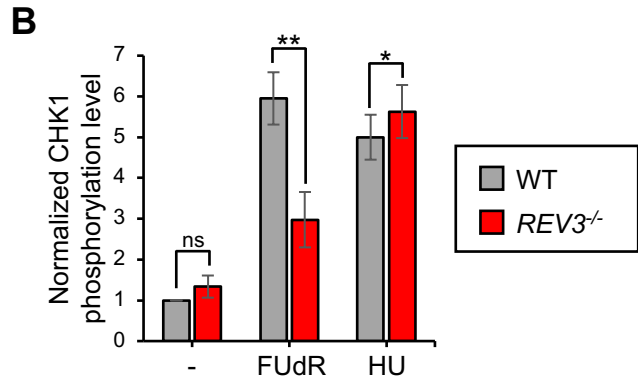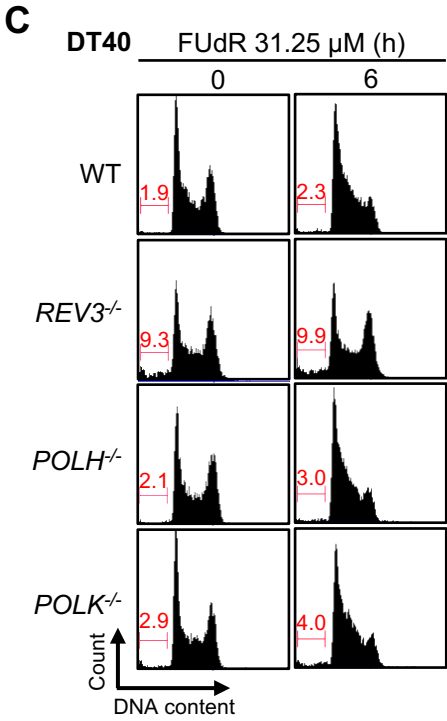

Supplement: S3 Fig — (A) Indicated TK6 cells were treated with 250 nM of FUdR or 50 μM of hydroxyurea (HU) for 9 h. Cell extracts were blotted for Chk1-pS345, Chk1, and GAPDH (loading control). (B) Quantification of Chk1 phosphorylation levels for indicated cells. Chk1-pS345 intensities were quantified and normalized to those of unmodified Chk1. The mean values of three independent experiments and SE were plotted. ns, not significant; *p < 0.05; **p < 0.01 (Student’s t-test). (C) Indicated DT40 cells were treated with 31.25 μM FUdR for designated times (0–6 h). The histogram presents the cell cycle distribution. The DNA content (stained with propidium iodide) is displayed on the x-axis on a linear scale, and the number of cells counted is presented on the y-axis. Two peaks at 0 h correspond to cells in the G1 (left) and G2 (right) phases. The numbers above brackets represent the percentage of cells in sub-G1 (dead cell fraction). (PDF) [file pgen.1011341.s003.pdf]
