# Supplementary material for: REV3 promotes cellular tolerance to 5-fluorodeoxyuridine by activating translesion DNA synthesis and intra-S checkpoint
Source: PLoS Genet. 2024 Jul 2;20(7):e1011341. doi: 10.1371/journal.pgen.1011341 (PMC11249241; doi:10.1371/journal.pgen.1011341)
Supplement: Attachment — Submitted filename: Rebutal letter0520(Mubasshir Wshif)_rk2kh3.docx [file pgen.1011341.s005.docx]

Reviewer #1: Review of: “REV3 promotes cellular tolerance to 5-fluorodeoxyuridine by activating translesion DNA synthesis and intra-S checkpoint”, by M. Wahif et al., submitted to PLOS Genetics.

The authors administer 5-fluorodeoxyuridine (5-dFUdR), a well-known anticancer an antiviral agent, to chicken or human cells and observe that cell variants lacking a functional REV3 enzyme survive more poorly and display several other defects relating to DNA replication, such as fork movement slowdown, chromosomal breaks and other DNA aberrations, altered cell-cycle progression, and loss of S1 checkpoint control. Overall, the authors conclude that REV3, encoding the catalytic polymerase unit of the established Translesion DNA-Synthesis polymerase Pol Zeta, plays a critical role not only in translesion synthesis but also in cell-cycle control and checkpoint activation after induction of DNA damage.
Overall, this work appears well performed and meaningfully interpreted. I have some minor concerns primarily on issues of presentation and explanation, as outlined below:
(Response) Thank you very much for your overall positive comments and constructive advice. We answered your points below.

1. The section on cell-cycle progression (pages 8-9 and Fig. 4) is hard to follow, especially for readers not directly familiar with cell sorting, etc. In Fig. 4A, what are the peaks, what are the read numbers above the brackets? (same for Fig. S2) Simply referring to ref. 4 for BrdU cell sorting is not sufficient. Needs better description.

(Response) Thank you for this constructive advice. We added the detailed descriptions on Figure legends for Fig. 4.

2. When you start exposing cells to FUdR what is the state of the cells? Are they actively growing? Please how cells were prepared for treatment.

(Response) We used actively growing cells. We described this information in the materials and methods section.

3. Are the results shown in Fig. 1 from plating experiments for surviving colonies? What did you use the ATP assay for? Please explain.

(Response) No. In Figure 1, we measured the number of living cells in liquid culture. To estimate the number of living cells, we used ATP assay. In this assay, we measured the concentration of ATP from living cells via the chemiluminescence of luciferase. We described this information in Figure legend for Fig. 1.

4. The FUdR sensitivity in Fig. 1A is based on a comparison of 90% survival data (Results first paragraph). However, looking at the actual survival data there are very few data point for this level of survival, at least for the most sensitive cells. Please explain how you did this.

(Response) We calculated the LD90 value which represents drug concentration making 10% of cells survive using GraphPad Prism 9 software from the survival curve (shown in S1 Fig.). This software estimates LD90 values by extrapolating the curve if the data do not include 10% survival data. This information was additionally described in the materials and methods section.

5. In Fig. 3B, explain the purpose of the red pointing triangles.

(Response) Thanks. The red arrowheads show the break point of the chromosome. We explained this in the figure legend in the revised manuscript.

6. In Fig. 5C, VE821 should be BE821 (or reverse)?

(Response) Thanks. We corrected this mistake in the Figure legend for Fig, 5C.

7. In Fig. 6, what are (exp) 1, 2 (are they duplicates? Please explain)

(Response) Thanks. Yes. Exp means the results of the independent experiment. We explained this in the figure legend in the revised manuscript.

8. Overall, the authors make strong statements on the role of REV3 in checkpoint activation for the case of FUdR. In view of certain effects that REV3 deletion seems to have on cell cycle events even in unexposed cells (various panels of Fig. 4), maybe some moderation may be appropriate. Also, one might comment on these effects as well. Might be relevant, as some of the effects in the deletion mutants could be indirect.

(Response) We agree to this comment and respectfully toned down in whole manuscript. We discussed the effect of the loss of REV3 in unexposed cells according to this comment.

9. In at least one case (page 14) I saw mMolar instead of microMolar. Please check carefully.

(Response) Thanks. We carefully checked and fixed the units (milli versus micro).

Reviewer #2: The authors analyzed the mechanism underlying cellular resistance to FUdR. They propose that REV3 promotes cellular tolerance to FUdR by activating TLS and the intra-S checkpoint. In particular, the conclusion regarding the intra-S checkpoint is unexpected. These data, revealing the mechanism of such an important biological reaction, should significantly contribute to this field. Furthermore, researchers not only in DNA repair (in Genetics) but also in the field of cancer research might be interested in this manuscript. Below are my suggestions for further strengthening the manuscript.
(Response) Thank you very much for your overall positive comments and constructive advice. We answered your points below.

Major:
1) REV3-/- DT40 cells showed considerably high sensitivity to FUdR (Figures 1A and C). However, REV3-/- TK6 cells exhibited only mild sensitivity to FuDR (Figure 1B). Thus, the effect of depleting Rev3 on FUdR sensitivity is different in DT40 cells compared to TK6 cells. The authors should discuss the reasons for this difference.

(Response) Thank you for this constructive advice. DT40 cells do not have p53 expression and higher expression level of Myc than human cell lines and thus the DNA damage checkpoint activity is reduced in DT40 cells and this cell line replicates their genome more rapidly than TK6 cells. The more prominent effect of FUdR on cellular survival in DT40 cells might be attributable to these cellular features. According to this comment, we discussed this point in the discussion part (2^nd^ paragraph).

2) In Figure 2, the rate of DNA synthesis in REV3-/- cells was significantly slower compared to that of wild-type cells after incubation with FUdR for 12 hours. Consequently, the authors concluded that REV3-mediated TLS maintains replication fork progression in the presence of 5-FdUMP and dUMP on template strands. However, the authors should exercise caution in interpreting these data. Since REV3-/- cells exhibit increased incorporation of dUMP and 5-FdUMP (Figure 6), it is challenging to attribute replication fork progression solely to the effect of TLS.
(Response) Thank you. We agreed with this comment and measured the effect of FUdR incorporated in the template strands in the other TLS mutant such as *POLH∆* cells which is proficient in intra-S checkpoint activation. We found that *POLH∆* cells also exhibited slowed replication on the FUdR-incorporated template. These data indicate that TLS function of Rev3 is responsible for the cellular tolerance to FUdR at least in part.

3) In Figure 3, REV3-/- cells exhibited more chromosome aberrations than the wild-type cells after treatment with FuDR. Additionally, Dr. Hirota reported that replication-blocking agents can cause chromosomal breaks unassociated with DSBs, specifically single-strand gaps left unreplicated (Ref40). Consequently, the authors need to discuss whether the chromosome aberrations induced by FuDR represent DSBs or unreplicated single-strand gaps.

(Response) Thank you for this constructive advice. We discussed this point in the revised manuscript (the fourth paragraph in the discussion part).

4) In Figures 4 and 5, it is noteworthy that Rev3 contributes to the activation of the intra-S checkpoint induced by FuDR. Conversely, Rev3 is not implicated in the activation of the intra-S checkpoint induced by HU in TK6 cells. The authors should verify the reproducibility of these findings using DT40 cells. Additionally, please discuss the different mechanisms of intra-S checkpoints induced by HU and FuDR, based on the results obtained by the authors.
(Response) We want to focus on the role of REV3 on FUdR-mediated replication checkpoint in this manuscript, and thus such additional experiments seemed to be beyond the scope of this study. Alternatively, we discussed the difference between HU- and FUdR-mediated replication checkpoints(the fifth paragraph in the discussion part).

Minor:
1) In Figure 3B, the image does not distinguish between isochromatid breaks and chromatid breaks. Therefore, it would be preferable to provide a clearer image. If presenting it clearly is challenging, it might be beneficial to include an illustration as well (e.g., Figure 1D and 4B in PMID: 21464321 [Yamamoto et al., PNAS 2010]).

(Response) Thank you for this constructive advice. We changed the representative images to clearer ones.

2) On page 10, line 3: "FudR" is incorrect.

(Response) Thank you. We fixed this error.

3) In Figures 4C and E, please conduct statistical analysis.

(Response) Thank you. We added the p-values to these data.

4) Is the UNG-comet method novel? If this method has already been developed, please provide a citation.

(Response) Thanks. This is not a novel assay. We additionally cited former studies.

Reviewer #3: Washif et al., provide evidence for a role of the translesion DNA synthesis polymerase REV3, a subunit of the TLS pol zeta heterodimer, in tolerance to 5-fluorodeoxyuridine(5FdU) in chicken DT40 cells. They identified Rev3 as important to tolerate 5FdU by analyzing a series of cell lines knock out for several genes involved in several DNA repair and DNA damage tolerance pathways. Rev3-/- cells stood out as the most sensitive cells to 5FdU. Authors showed that Rev3-/- cells reduce replication fork speed compared to wild-type cells upon 5FdU treatment and that these cells also were deficient in activating the ATR-dependent checkpoint thus entering mitosis with stretches of unreplicated DNA, resulting in chromosome breakage. These studies are consistent with the role of TLS in tolerating the insertion of 5FdU as previously shown by the authors for the second Pol zeta subuinit REV7, however a role for REV3 in activating the ATR-dependent checkpoint was not previously reported and constitutes the most original part of this work which is of general interest. However, this part is not much developed, and to my opinion, it warrants further investigation in clarifying the role of REV3 in ATR-dependent checkpoint activation, and obtaining some evidence that a similar process may also occur in a mammalian cell line. Below are the main key points that if addressed in my opinion would make this work a strong candidate for publication.
(Response) Thank you very much for your constructive comments and advice. We answered your points below.

1. While DT40 chicken cells are an interesting and pertinent genetic model, for this work to be of general interest, it would be important to determine whether also mammalian cells knock out for REV3 show a similar sensitivity to 5FdU, compared to knock out of at least another TLS pol.

(Response) Thank you. We additionally analyzed the *REV1*^-/-^ , *POLH*^-/-^, and *POLK^-/-^* TK6 for the cellular sensitivity to FUdR and newly shown in Fig 1D.

2. It is known that Pol zeta makes a quaternary complex with Rev1 and Polk (Wojtaszek J et al., JBC 2012; Xie et al., Protein Cell 2012). Interestingly, two previous reports have shown that activation of the ATR-dependent checkpoint is compromised in the absence of either Polk or Rev1 in both Xenopus egg extracts and mammalian cells (Bétous et al., EMBO J 2013; DeStephanis et al., BBRC 2015). Hence, one may wonder whether the inability to phosphorylate Chk1 in REV3-/- cells could be due to destabilization of this complex and/or reduced gene expression of ReV1 or Polk. Also, Fig. 5A shows that the total level of Chk1 is also reduced upon REV3 knock out. A more recent report has provided evidence for Chk1 stabilization by Polk (Dall’Osto et al., MCB 2021). Hence, I believe that this point required further investigation in order to clarify whether the checkpoint activation function of Rev3 may be in concert with that of Rev1 and/or Polk.

(Response) Thank you for your constructive comments. We analyzed the REV1-/- and PolK-/- DT40 cells for their ability to induce intra-S checkpoint upon FUdR treatment and showed that REV1 and REV3 but not Polk are required for checkpoint activation upon FUdR. These results were consistent with the stronger FUdR sensitivity of *REV1*^-/-^ and *REV3*^-/-^ cells than that of other TLS mutants including *POLH^-/-^* and *POLK*^-/-^ cells and indicate that Polζ plays a critical role in checkpoint activation upon the replication stress induced by FUdR without requiring Polκ. These new data are shown in S2 and S3 Figs.

3. Another important point is to speculate how 5FdU is dealt with in DT40 cells. Authors show that Rev3-/- cells are much more sensitive to 5FdU than cells bearing a point mutation in the PCNA lysine residue (K164R mutation) ubiquitinated by Rad18, that allows Y-family TLS pols recruitment. Now, neither Polzeta nor Rev1 require PCNAmUb to be recruited. However, Rad18-/- cells are much more sensitive to 5FdU than PCNAk164R. How the authors explain this difference? Then, in terms of TLS function, what can be draw from these results? Is 5FdU TLS on the fly in DT40 cells? This could explain results shown in Fig. 2. Fiber spreading assay in mammalian cells Rev3-/- could be interesting to determine whether what observed by the authors is specific to DT40 cells. In fact, “on the fly TLS” by Rev1 has only been observed in DT40 cells (Edmunds et al., Mol Cell 2008).
(Response) Thank you. Rad18 might have wider roles in the maintenance of the genome than PCNA ubiquitination since Rad18 avoids toxic NHEJ of DSBs caused during DNA synthesis to prevent abnormal chromosomal fusions (Kobayashi S. Oncogene 2015). We agree with the comment that we need to address the conserved role of Rev3 in TLS on FUdR-damaged templates in human TK6 cells. We showed that *REV3*^-/-^ TK6 cells also showed slowed replication fork progression on FUdR-damaged templates.

Other points
Figure 1, 4B-E, 5C and S1 miss significance tests. Fig. S1 is very crowed, maybe it can be split in several panels so to better read and ascertain the significance of the differences.

(Response) Thanks. We added the p-values in these data. The S1 Fig. was split in three panels.
