# Supplementary material for: REV3 promotes cellular tolerance to 5-fluorodeoxyuridine by activating translesion DNA synthesis and intra-S checkpoint
Source: PLoS Genet. 2024 Jul 2;20(7):e1011341. doi: 10.1371/journal.pgen.1011341 (PMC11249241; doi:10.1371/journal.pgen.1011341)
Supplement: Attachment — Submitted filename: Response to reviewers.docx [file pgen.1011341.s006.docx]

Response to reviewers

Reviewer #1: Comments were addressed appropriately.

(Response) Thank you so much for your constructive comments. With your critical help, our manuscript was improved.

Reviewer #2: The authors thoroughly addressed all concerns. I recommend to publish this paper in PLOS Genetics.

(Response) Thank you so much for your constructive comments. With your critical help, our manuscript was improved.

Reviewer #3: While the authors have now improved the manuscript, there are still few issues that need to be resolved in order to strengthen the conclusions, listed here below.

(Response) Thank you so much for your constructive comments. We described our response to your comments below.

1. It is clear from figure 5 and Figure S3 that REV3 deletion affects the stability of Chk1. This observation has to be taken into account in the interpretation of the results.
(Response) Thank you for this constructive comment. We added the descriptions in the fifth paragraph of Discussion (page 12, line 22) as follows. “Since we observed a slight decrease in Chk1 protein levels in DT40 *REV3^-/-^* cells (Fig 5A) and TK6 *REV3^-/-^* cells (S3 Fig), it is possible that REV3 contributes to the faithful checkpoint activation through maintaining Chk1 protein stability at least partly.”

2. In response to the different sensitivity of PCNAK164R mutant cells compared to Rad18-deleted cells, although its is known that Rad18 is also involved in DNA double strand break repair, I do not see how this function fits with the sensitivity to 5FdU. Maybe the authors think to a possible role of Rad18 in repairing gaps generated by 5FdU in REV3-deleted cells by promoting homologous recombination? (See also comments below). Further, authors have not provided explanation nor additional data about “on the fly” TLS in the presence of 5FdU.
(Response) Thank you for this constructive comment. We thought that Rad18 is involved in the processing of DSBs caused by fork collapse by FUdR. We added a new paragraph to explain the possible roles of Rad18 besides its role to activate TLS through the ubiquitination of PCNA (sixth paragraph in discussion part, page 13, line 17~) as follows.

“We demonstrated that *RAD18^-/-^* cells exhibited significantly higher sensitivity to FUdR than *PCNA^-/K164R^* cells (Fig 1C). Since Rad18 induces PCNA ubiquitination at K164 and activates TLS [53, 67], these different FUdR-sensitivities between *RAD18^-/-^* and *PCNA^-/K164R^* cells were unexpected and suggested possible roles of Rad18 besides its role to activate TLS. A possible rationale is that Rad18 might avoid the toxic NHEJ leading to chromosome fusion. As FUdR affects DNA replication (Fig 2), DSBs might be induced due to fork collapse and such DSBs can be repaired by either HR or NHEJ. But one-ended DSB caused during DNA synthesis should be exclusively repaired by HR and NHEJ-mediated repair leading to detrimental chromosome fusion is strictly suppressed by Rad18 [35]. Another possible hypothesis is that Rad18 contributes to cellular tolerance to FUdR by inducing break-induced replication (BIR)-like DNA synthesis at the stalled replication fork [68]. Rad18 is recruited to ubiquitinated histone H2AX upon HU and induces DNA synthesis in a manner that requires BIR factors including PolD3 [68]. This view was supported by the observation that *POLD3^-/-^* exhibited higher FUdR-sensitivity than *PCNA^-/K164R^* cells like *RAD18^-/-^* cells (Fig 1C).”

3. Figure S1 still misses statistical significance tests.
(Response) We added the results of statistical significance test in S1 Fig.

4. Discussion, authors provide an explanation for the different effect of 5FdU compared to hydroxyurea (HU) treatment which is unclear to me. In particular I do not see what is the difference between “the entire replisome” and “individual replisomes” as mentioned by the authors. Maybe the authors intend that 5FdU does not arrests all replication forks? If this is the case, I do not see why. As far as I am aware, 5FdU as a milder effect than HU on nucleotide synthesis, since it interferes with the synthesis of the dTTP nucleotide and also promotes dUTP accumulation. This probably generates discontinuous DNA synthesis at all replisomes generating single stranded (ss)DNA gaps. In contrast, HU interferes with the synthesis of all nucleotides leading to arrest of the DNA synthesis process and generation of long stretches of ssDNA since the DNA helicase component of the replisome is not affected. The canonical ATR-Chk1 pathway does depends upon the 9-1-1 complex for activation. Probably the more likely explanation is that Chk1 activation in REV3-deleted cells occurs at ssDNA gaps that are left behind the replication fork? Here the authors discover that REV3 is important to activate the ATR-dependent checkpoint, probably by recruiting the 9-1-1 complex at ssDNA post-replicative DNA gaps.

(Response) Thank you so much. We agree with your opinion and changed the description in the fifth part of Discussion (page 13, line 5~) as follows.

“Since HU interferes with the synthesis of all nucleotides leading to the arrest of the DNA synthesis process and generation of long stretches of ssDNA [22], such HU-mediated long stretches of ssDNA might efficiently activate canonical ATR-Chk1 pathway. In contrast, FUdR interferes with the synthesis of the dTTP nucleotide and also promotes dUTP accumulation [28-33], and then resulting in the discontinuous DNA synthesis at all replisomes generating single stranded (ss)DNA gaps. Since the 9-1-1 alternative checkpoint clamp comprising Hus1, Rad1, and Rad9 physically interacts with REV7 [66], a possible rationale is that Polζ recruits 9-1-1 complex to such ssDNA gaps to transmit DNA damage signal to Chk1.”
